# Topic Modeling and User Network Analysis on Twitter during World Lupus Awareness Day

**DOI:** 10.3390/ijerph17155440

**Published:** 2020-07-28

**Authors:** Salvatore Pirri, Valentina Lorenzoni, Gianni Andreozzi, Marta Mosca, Giuseppe Turchetti

**Affiliations:** 1 Institute of Management, Scuola Superiore Sant’Anna, 56127 Pisa, Italy; v.lorenzoni@santannapisa.it (V.L.); g.andreozzi@santannapisa.it (G.A.); giuseppe.turchetti@santannapisa.it (G.T.); 2 Rheumatology Unit, Department of Clinical and Experimental Medicine, Università di Pisa, 56126 Pisa, Italy; marta.mosca@med.unipi.it

**Keywords:** social media, Twitter, systemic lupus erythematosus (SLE), network analysis, topic modeling, text analysis

## Abstract

Twitter is increasingly used by individuals and organizations to broadcast their feelings and practices, providing access to samples of spontaneously expressed opinions on all sorts of themes. Social media offers an additional source of data to unlock information supporting new insights disclosures, particularly for public health purposes. Systemic lupus erythematosus (SLE) is a complex, systemic autoimmune disease that remains a major challenge in therapeutic diagnostic and treatment management. When supporting patients with such a complex disease, sharing information through social media can play an important role in creating better healthcare services. This study explores the nature of topics posted by users and organizations on Twitter during world Lupus day to extract latent topics that occur in tweet texts and to identify what information is most commonly discussed among users. We identified online influencers and opinion leaders who discussed different topics. During this analysis, we found two different types of influencers that employed different narratives about the communities they belong to. Therefore, this study identifies hidden information for healthcare decision-makers and provides a detailed model of the implications for healthcare organizations to detect, understand, and define hidden content behind large collections of text.

## 1. Introduction

In recent years, the way in which researchers’ results, discoveries, and knowledge have been disseminated has changed significantly. The advancement of Internet technology has enabled the rise of social media platforms such as Facebook, Twitter, Reddit, and others to serve as channels where people interact, share opinions, and debate. These forums create communities where people establish relationships and interactions among themselves.

These online communities can influence and can be influenced by other online communities. This spread of influence plays a major role in the spreading of information, some of which may affect people’s offline behavior [1].

Content produced on social media can spread quickly throughout these communities, triggering rumors and cascading effects that can deeply influence political decisions, economic choices, social well-being, perceptions, and beliefs [2].

The use of social media text analysis and social network detection is not new in the public health field. Many studies have investigated the areas of forecasting clinical surveillance [3,4] and misinformation within and across health communities [5]. These studies contain considerable evidence suggesting that technology has been useful in the health domain, generating considerable awareness on social media, and helping people who live in remote areas [6] or who have little access to treatment [7].

Most of these studies have focused on epidemic and infectious diseases, while in the field of chronic diseases efforts have been mainly devoted to well-known diseases like diabetes or cardiovascular disease [8]. To our knowledge, little effort has been made to investigate the online communities’ dynamics around rare and complex rheumatic diseases, such as systemic lupus erythematosus (SLE), which is a chronic autoimmune disease whose management is still challenging due to the variety and complexity of the symptoms. These challenges greatly impact SLE patients’ quality of life and social activities [9]. Additionally, SLE also faces significant and complex unmet needs that must be dealt with [10], such as diagnostic delay and high burden of therapy [11], which puts pressure on healthcare costs.

Despite this lack of deep investigation of the social media interaction phenomenon for this complex rheumatic disease, patient associations, healthcare communities, blog pages, and patients are active on social media in order to seek information and increase awareness among the general public. In most cases, patients use these channels for emotional and peer health support [12,13], often searching for new treatments or healthcare decision suggestions [14].

### Literature Review

Literature on social media analysis has been previously analyzed in different applications that explore the pivotal role played by people’s perspective and community interactions to obtain worthwhile information for healthcare decision-making [15]. Applications of social media analysis for collecting information on behavioral patterns have previously been proposed under different conditions and with different purposes. In cancer, for instance, content analysis of discussions related to medication use and side effects [16] showed how the internet can be a valuable way for individuals to report side effects, and how healthcare professionals can support an effective medication adherence plan by monitoring the social media discussion. Another example can be found in tweets about diabetes and diets [17], emphasizing how some users acting as diabetes advocates can spread information and serve as opinion leaders, thus influencing others’ attitudes and behavior [18].

Other studies have reported the beneficial effects of higher patient satisfaction and patient engagement when hospitals create valuable social media interaction and strategy, providing better value for the hospitals adopting such a policies [19].

A recent literature review [20] that explored the effects of social media interaction on patient and healthcare professional relationships pointed out how patients mainly use social media for social support, which is represented through information support, emotional support, esteem support, and network support.

One of the main advantages of Twitter is the fact that users can express themselves freely, reducing the bias effect that often affects other types of investigation methods, such as online surveys or interviews [21]. On the other hand, it is important to consider the risks of using Twitter in social and healthcare research given the unrepresentativeness of the user community, the spread of misinformation, and difficulties in verifying the credibility of sources.

However, we believe that perspectives and views held by community members and expressed on social media platforms represent a good proxy of feelings and attitudes that might influence decision-making of other communities or users. Identifying as precisely as possible the content of these feelings and attitudes would improve the development of a tailored strategy for public health issues.

Analyzing the network dynamics and the role played by key users in the network community (such as influencers and opinion leaders) offers a gatekeeping tool to understand how information enters, flows, and spreads throughout the communities, and who drives it.

## 2. Methods

The objectives of this study were (1) to investigate and identify the common themes that spread on Twitter during World Lupus Day and to (2) detect the communities’ network dynamics, identifying “influencers” and their communities’ features.

### 2.1. Proposed Methodology

Using Twitter public streaming API, tweets released on the 10th of May 2019 containing at least one of the following words or hashtags were collected and analyzed: #WorldLupusDay, #lupus, #SystemicLupusErythematosus, or #SLE. A total number of 4434 (including retweets) tweets took into account information (i.e., time, location, sources, retweets, retweet count, follower count, and friend count) were collected. Tweets came from 2813 unique users. R software was used for the analyses.

**Figure 1:**
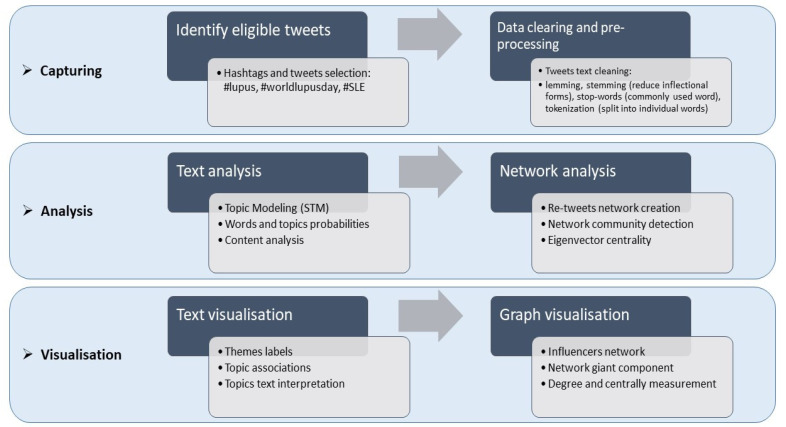
Framework workflow of social media Twitter analysis.

A comprehensive analysis flow is presented in Figure 1. Following the scheme of social media analytics [22], it is possible to extract patterns, discover hidden information, and outline network interactions among online communities by mining the health discussions.

In stage one (capturing), we collected tweet texts and information containing keywords or hashtags released on Lupus Day through the Twitter API. Next, data-cleaning and pre-processing were applied to the entire dataset obtained. In stage two, we performed data analysis using two main techniques: (a) text analysis/natural processing languages through word frequencies, n-gram, and topic modeling, and (b) network analysis and measurements (statistics and scores of the network under investigation). Stage three focused on results visualization. Visualization techniques, such as bar-charts, histograms, network graphs, and other visualization types, assumed a key role in interpreting and presenting results.

### 2.2. Data Cleaning and Pre-Processing

Data were gathered to employ retweet [23] packages belonging in the R software. On the basis of data collected, the influencer score and network influence score were calculated. The influencer score represents a proxy to identify the small percentage of users who have a large connection (followers) to a large audience who follow them and have established a sort of trust in which their posted content creates perceived influence [24]. On the other hand, the network influence score, which is based on the number of retweets received by other users, represents a sort of endorsement of a specific content or message shared. The further a tweet spreads, the more influence the user has.

We can summarize the two scores by saying that the first score is more oriented toward the enormous attraction of followers one is able to obtain based on shared lifestyles, opinions, and textual content [25]. The second score is more based on the attention and endorsement that a tweet content (or a set of tweets) is able to achieve, being shared throughout a user network in a certain span of time [26].

Despite the efforts and increasing interest in properly measuring and assessing an influencer’s score, when detecting a user’s ability to maximize and spread content and thus shape followers’ perceptions and behavior there is still a clear lack of widely recognized measures that are able to do so [27]. Nevertheless, some studies, especially from marketing literature [28,29], have developed robust measures to gain solid proxies of the social media influencers’ effect. In our study, we obtained the influencers score, aggregating the performance of Twitter indicators addressed by Anger Isabel and Kittl Christian [30]. The score index was calculated as the average of the sum of three different ratios: the ratio between the number of followers over the number of following (R_f_); the retweets and mention ratio (R_rt_), which is calculated as total retweet count over the total number of tweets created; and the interaction ratio (R_i_) obtained dividing total retweets count by the number of followers. The aggregation of three independent ratios reduced the possibility of misinterpretation based on the mass-followers effect. Nevertheless, it is important to keep in mind that other measures exist, which could integrate even more sophisticated scores [29].

### 2.3. Network Analysis

The scoring index for the network influence score (ii) takes into account typical approaches from social network analysis, which considers independent indexes from graph theory [31], i.e., betweenness centrality, out-degree, PageRank, and others. To detect influencer users in the network dynamics, we considered retweets as a proxy to represent an endorsement to the tweet content shared by the user. The modularity [32] detection algorithm was employed to identify communities (clusters) that compose retweet network. Basically, the modularity algorithm divides a network into a set of clusters where each node (user) belongs to only one cluster. It measures the strength of the identified clusters in the network where modularity group nodes exhibit high density with each other. The Force Atlas 2 [33] algorithm was employed to visualize the network layout. It is a force-based algorithm that draws linked nodes closer while pushing unrelated nodes farther, addressing hubs in clusters. This visualization provides a readable representation of the entire graph.

As a score index, the eigenvector centrality [34] was employed to determine the influencer nodes. Eigenvector centrality is a measure of the node’s importance in the entire network weighed on the nodes’ connection. For our purposes, this was the most suitable index to identify influencer nodes [35]. To calculate and compute the network analysis, score, and visualizations, we used Gephi software [36].

### 2.4. Text Analysis and Topic Modeling

Topic modeling is a branch of unsupervised methodology for the natural processing language applied to analyze and extract topics from a corpus of documents. This approach fit the text analysis for Twitter content quite well. Considering, the unsupervised nature of the topic modelling method, it was possible to identify the thematic structure (topics) within the set of tweet texts without any prior data manipulation, like text-labeling or training dataset. Topic modeling application allowed the discovery of the thematic structure in a large corpus of text, making it possible to organize, summarize, and visualize the latent themes and patterns present in any kind of text corpus [37].

**Figure A1:**
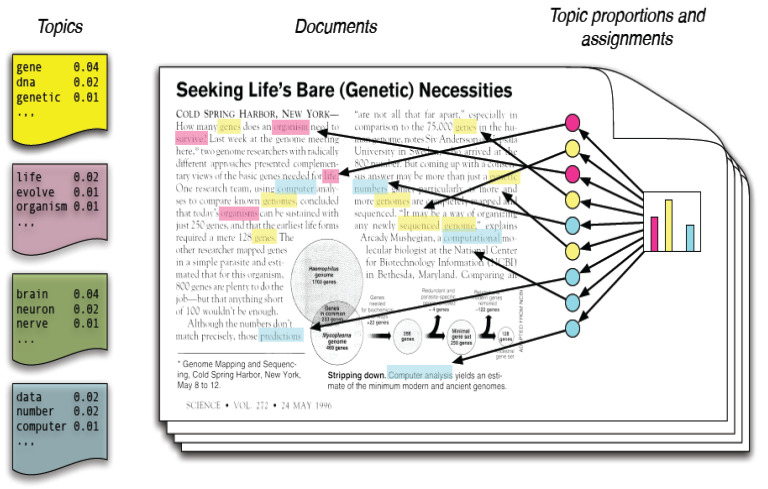
Source: David M. Blei. “Probabilistic topic models”. Communications of the ACM (Association for Computing Machinery); 2012, Vol. 55 No. 4, Pages 77–84. 10.1145/2133806.2133826.

The most common topic modeling approach used was the latent Dirichlet allocation (LDA) [38], which is a generative probabilistic model assuming that a document is composed of a set of (latent) topics, where each topic is composed of a set of words. This approach can be thought of as a classification method instead of a numerical feature or collection of words one could group together in a meaningful way. See Figure A1 in the Appendix A for more details.

**Figure A2:**
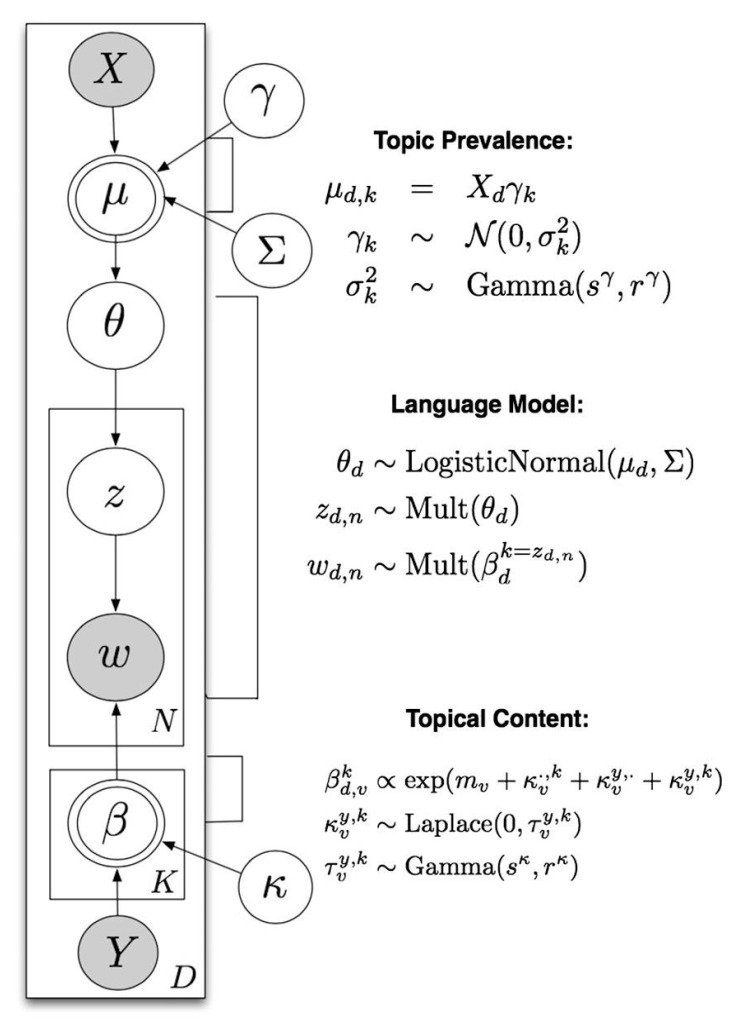
Structural topic modeling, in plate notation, in: (Roberts ME, Stewart BM, Tingley D, Airoldi EM. The structural topic model and Applied Social Science 2013).

A recent application that can expand the ability of the LDA framework to gain valuable results from a large corpus of text is structured topic modeling (STM) [39]. STM provides the possibility of considering metadata associated with the text, such as the author of the tweet, the associated numerical score, and other characteristics of the overall dataset using document-level covariates. After identification of the latent topic, using the stm R package [40], we estimated the effect influencer score and network influencer score as covariates had on topic prevalence, exploring whether and which topics had a higher probability of appearing in tweet texts, aiming to investigate whether different topics were used in different ways. See Figure A2 in the Appendix A.

## 3. Results

From the dataset composed of 4434 tweets, a network to analyze the network influencer score was created involving 2813 unique users and employing a direct graph. Each node represented a user and the edge between two nodes was established when a user’s tweet was retweeted. We considered the giant component network and the smaller disconnected components were dropped out (18.3%). More details on the network analysis are provided in the Appendix A. See Figure A6 and Figure A7.

**Figure 2:**
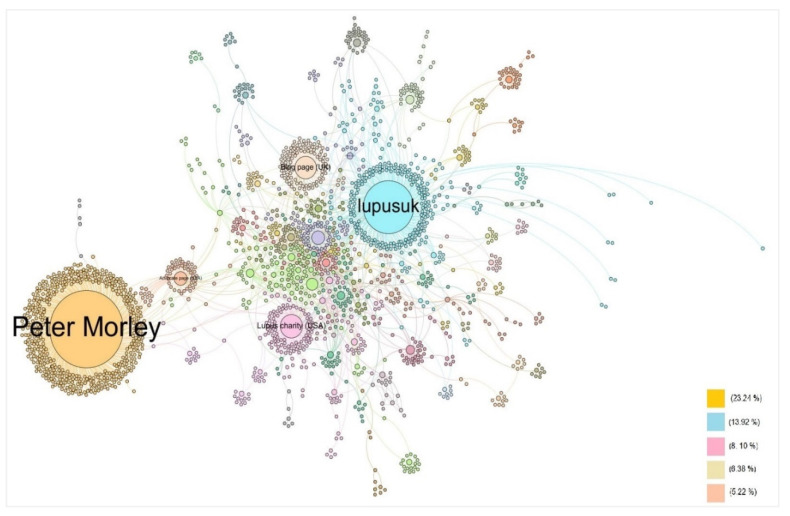
Retweet network analysis.

The size of the nodes was proportional to the number of social connections based on the number of retweets a specific user received. Nodes and edges had the same color if they were linked to each other, making the detection of communities possible. The node position in the network was determined by a heuristic that attempted to locate nodes connected closer together, which thus revealed the communities’ structure. See Figure 2.

The community detection algorithm found 25 communities (clusters). The top five communities accounted for more than 55% of all network connections. Applying the eigenvector centrality algorithm to detect the most influential users, five nodes emerged as the most influential. These five users received more attention, intended as the number of retweets, allowing them to catalyze a vast amount of attention based on their tweet text content shared. We asked the top influencers identified for their permission to display their account name. Four of them consented to display their names; for the others, we used anonymized acronyms to identify the account type.

As reported in Table 1, only one account appeared in both influencer scores. This was due to the fact that the two scores were intended to measure different dynamics. Nevertheless, considering the specificity of the dataset collected, in this case it was also true that two different types of influencers played a different role and showed different features in attracting attention based on their posted content. Interestingly, the highest scored user was Peter Morley, whose network is weakly connected with the rest of the main users’ connections. He is easily visible in Figure 2 with his peripheral position in the network structure.

After the influencer score analysis and the network relationship measurement, tweet text analysis was employed. We adopted STM on the entire tweet text dataset.

**Figure A3:**
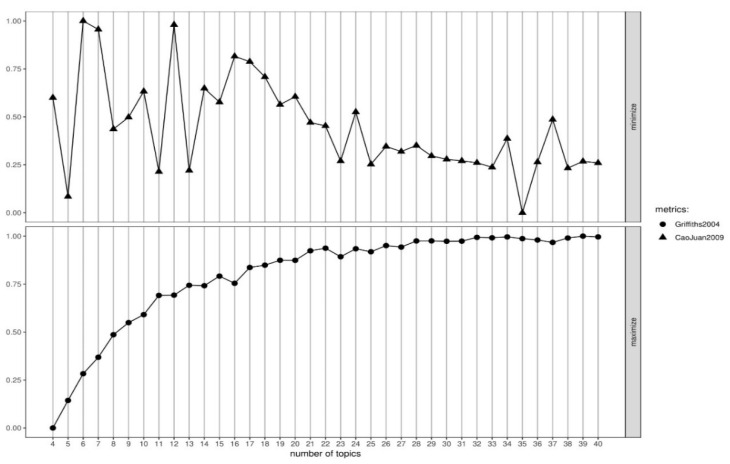
Find optimal number of topics.

When performing STM, several steps need to be addressed before reaching the final evaluation, including the identification of the proper number of topics (k) that better represents the number of themes in the text corpus. Different approaches exist; no one is more correct than the others. In our analysis, we based the optimal number of latent topic on “Griffiths” [41] and “CaoJuan” [42], which are metric scores implemented in the ldatuning package [43] that use the log-likelihood method via Gibbs sampling. Griffiths metrics maximize likelihood, while CaoJuan metrics minimize divergence between topics. As a result, the optimal number of topics (k) for our dataset was 12 topics. In the Appendix A, Figure A3, the optimal number of topic plots is provided.

**Figure A4:**
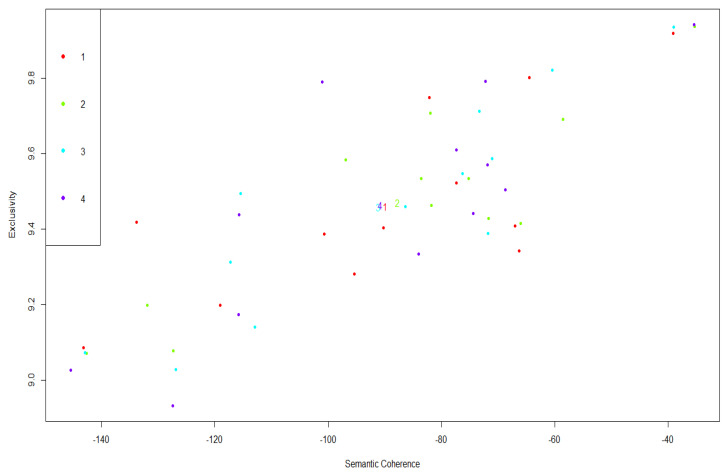
Topic models selection in STM packages.

**Figure A5:**
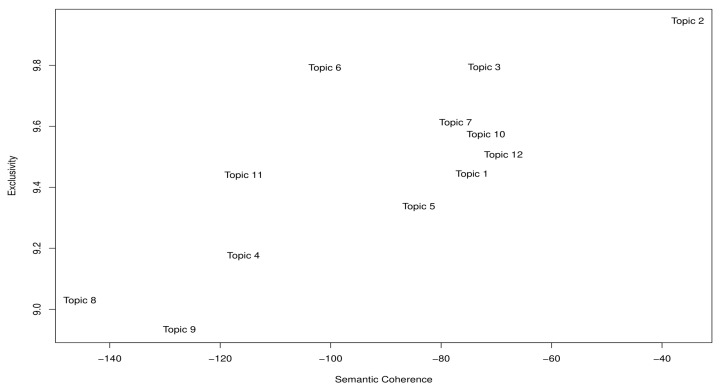
Plots result pf the selected model semantic coherence and exclusivity for each 12 topics.

**Figure A6:**
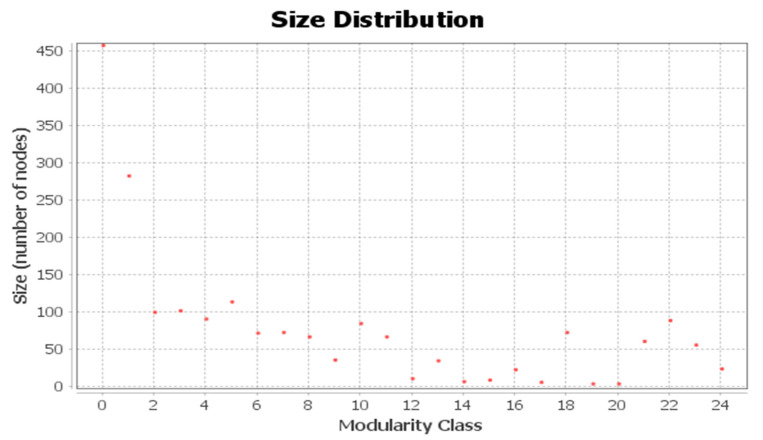
Community size distribution.

**Figure A7:**
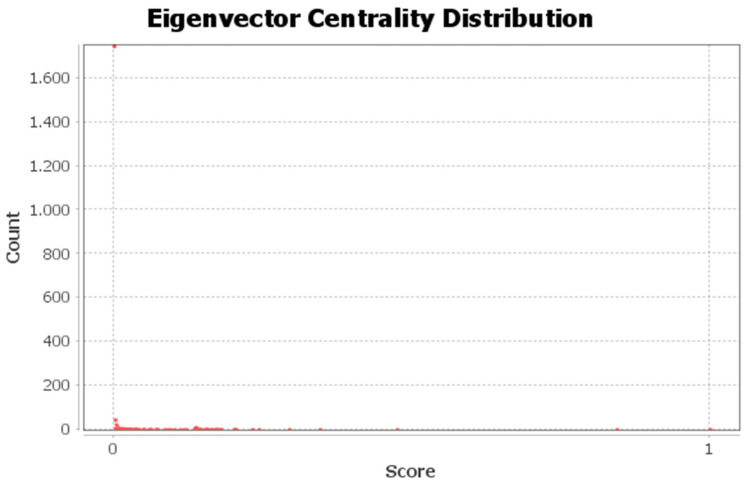
Eigenvector distribution of retweet network data.

Another step in the STM that needs to be addressed before reaching the final evaluation is the choice of the model that best estimates the possible outcome. There are different initialization parameters that need to be evaluated, discarding models with low likelihood values [40]. Even in this case, there is no ground truth approach. However, assessing the quality of the models by considering the trade-off between semantic coherence [44] and exclusivity [39] for each topic within the model is one of the most suitable approaches. The semantic coherence metric is related to pointwise mutual information that measures the most probable words in a specific topic that occur together. The exclusivity measure includes information on word frequency employed in the FREX metric [45]. These measures provide the distinctness of the topics, making possible a comparison of the highest scores, ensuring the quality of the model selected. Plots and results of the selected model are provided in the Appendix A Figure A4 and Figure A5.

**Figure 3:**
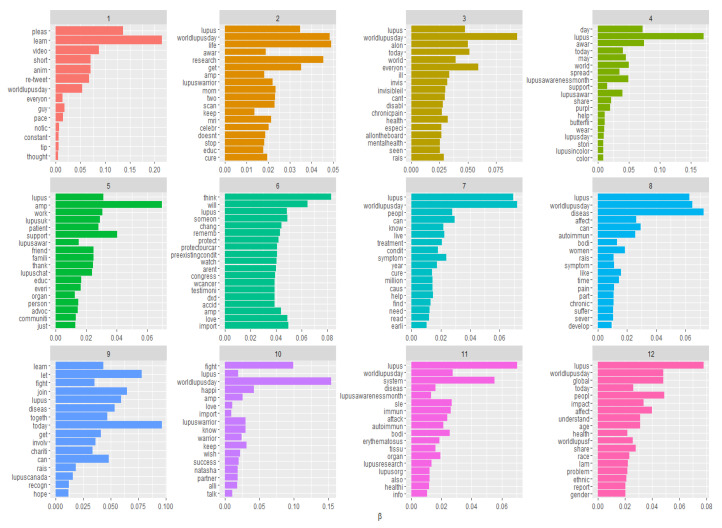
Topics and themes identified in the tweet text corpus.

The results of the topic model are shown in Figure 3. Specific words were linked to specific topics accordingly with their (beta) β probabilities of belonging to the topic. Topic labels were not automatically generated. Label selection was the moment when researchers analyzed the results after the parameter setting to check what emerged from the model’s execution, and to decide whether the emerged allocation was coherent, or if more model executions were needed. In our case, for each topic a specific label was identified using the authors’ judgment obtained through an open discussion until a consensus was reached. Indeed, topics were interpreted and labelled on the basis of the probability of each word belonging to each specific topic.

In doing so, we also checked the most representative tweets related to the topics, to better understand the meaning of the topics by inspecting highly correlated tweet texts. A sample of the topics and the associated tweets are reported in Table 2.

From the topic model results, clearly latent themes behind the tweet texts discussion emerged, underlining a hidden structure that aimed to share something more than just awareness messages or informative content. Some topics that emerged appeared to be similar yet still covered different issues and tackled different narratives, which attracted the attention of different users. To capture the effects that different topics may have on different types of users, we employed a measurement of the covariate impact. As previously mentioned, the main difference between the LDA and STM is the possibility of incorporating metadata and estimating the relationship between the selected covariates and the topics [40].

**Figure 4:**
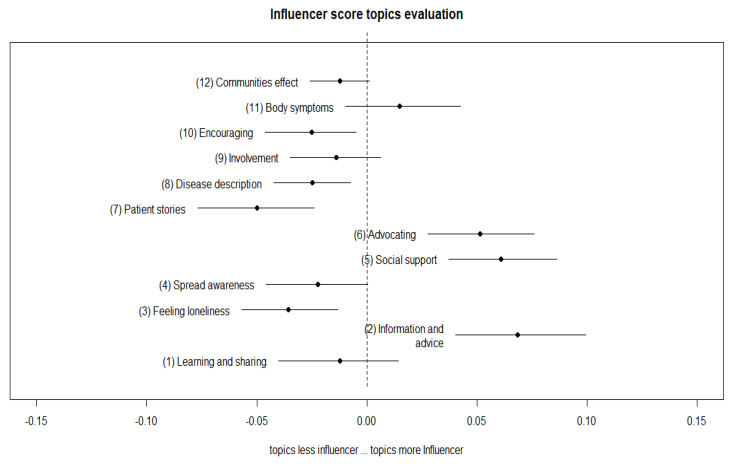
Estimated topic proportion to be discussed by influencer score.

**Figure 5:**
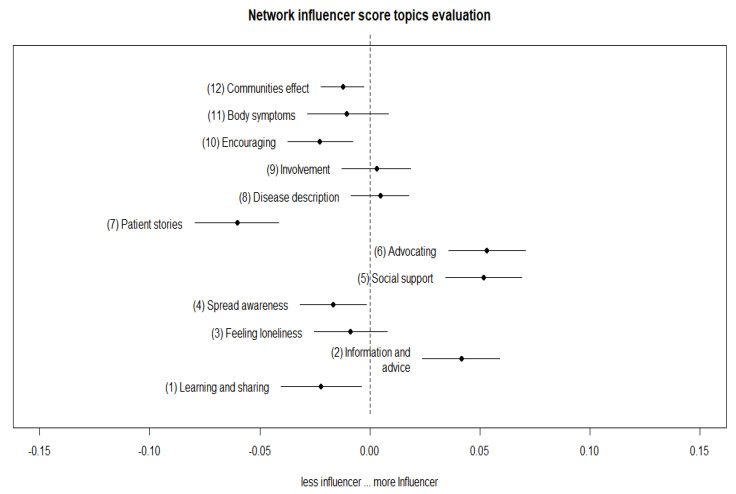
Estimated topic proportion to be discussed by network influencer.

Figure 4 and Figure 5 show the estimated proportion of topics more likely to be used and discussed according to the value of influencer score and network influencer score in the contents of their tweets. Topics whose estimates lie on the right side (corresponding to positive values of the *x*-axis) were more likely to be discussed/used by influencer, and conversely for the left side.

Such an approach made it possible to evaluate the uncertainty surrounding the coefficient, performing a regression where the topic-proportions were the outcome variable, based on the covariance matrix. The results allowed the estimation of topic proportion as a function of covariate data, which further produces confidence intervals around the estimated topic [39].

Interestingly, the results of the estimated topic prevalence showed that some topics and their prevalence were different between the two types of influencer. In particular, topic number 6, the advocacy theme, was largely associated with the network influencer tweet content. We assumed that this kind of topic and the related discussion attracted an enormous amount of attention from a specific type of user related to network influencers. In other words, it was more probable that the topic was related with advocates’ content, i.e., in favor of new policy law, health policy attention, or in support of specific collective actions. This can attract specific attention and spread the narrative under discussion faster and more deeply in specific communities.

Topics 8 (disease description) and 9 (involvement) received less attention from the general public and were more likely to occur in the influencers’ network communities, which may be more attracted to news or information about possible new treatments or sustaining program involvement.

Instead, topic 11 (body symptoms description) was more likely to receive attention from general influencers. Thus, the public was more interested in understanding the illness and its manifestations.

**Figure 6:**
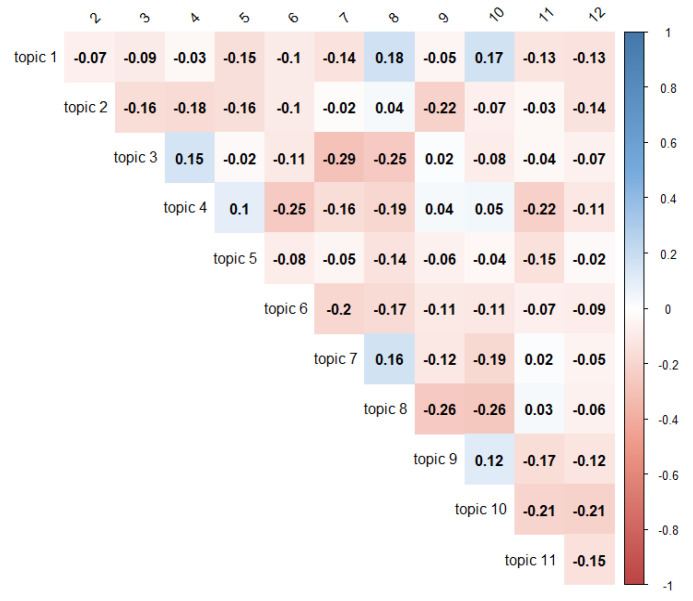
Correlation topics matrix.

The STM also allowed an exploration of the correlations between topics to evaluate topics more likely to be discussed in the same tweet. Figure 6 shows pairwise correlation coefficients between identified topics. Positive correlations (in blue) indicate that both topics were more likely to be discussed in a tweet, and vice versa for the negative correlation coefficient (in red). A positive correlation appeared between topics 1 and topic 8, addressing discussions about the disease description and the way in which it was possible to learn and share information on SLE.

A fairly negative correlation appeared between topics 3 and topic 7, which referred to patient stories and loneliness. It is our opinion that these two topics were less likely to be discussed in a tweet together because patient stories tended to describe the illness’ physical symptoms, while tweets about loneliness were more a consequence of the disease and tended to be oriented as messages in order to feel less alone. However, as previously mentioned, there was no positive or negative correlation in our results, so we did not have enough information to make more assumptions. Further research could explore more deeply how topics are related and discussed with each other and evolve over time.

## 4. Discussion

Twitter enables millions of users to share information worldwide in real time. This phenomenon allows policymakers, healthcare stakeholders, and other people to influence and be influenced by opinions and discussions that flow across online social communities, making it possible to share valuable information and practices more quickly and easily than ever before. Such a possibility has become a rich source of value for information-gathering and practical dissemination, in particular for complex and low-intensity diseases like Lupus [46].

Interactivity among online communities makes it possible to renovate not only the healthcare organizations’ online approach but the manner in which people’s attitudes and intentions regarding health behavior might be influenced [47]. However, valuable information is complex to detect and depict, considering how vast and fast social media platforms work, too often spreading rumors or misinformation [48].

For these reasons, investigating the dynamics played by online communities during specific events like the World Lupus Day can offer a powerful tool to stakeholders for identifying and setting up policy strategies for gathering valuable information and sharing good practices. This ability can offer a concrete tool for decision-makers in dealing with information asymmetry [49], obtain valuable new elements for the decision-making process, promote trust across the identified communities, and promote health-seeking behavior [50].

In our study, we sought to analyze latent themes spread on Twitter during World Lupus Day and detect online user communities’ behavior by measuring the users’ retweet network.

We measured and found two different types of influencers in our analysis, who behaved and acted differently. There was one type of influencer who was more generally public-oriented, measured on the ratio between the number of followers and the ability to amplify the content they posted, and a second type of influencer, more based on the retweets and network attention count, as an endorsement of their tweet content.

Network influencer users, mostly led by patient organizations, have many followers who tend to have intense connections among themselves, and show more interest in specific topic discussions about the role of social support and policy advocacy. General influencers show less network connection and appear to attract more followers with content related to general disease advice.

Many topics discussed by the two types of influencer were in common. However, the attention posed in some topics were different. This is well represented by the discussion order of topics 2 (Information and advice), 5 (social support), and 6 (advocating), which are swapped in the likelihood order.

Another difference between the topics was posed by the fact that general influencers discussed body symptoms (topic 11), whereas the network influencer discussed topics related to patient involvement (topic 9) and diseases description (topic 8).

To the best of our knowledge, this study is the first to employ a combination of methods to explore deeply latent topic discussions and online communities’ interactions regarding a low-prevalence disease like Lupus. Unlike other kinds of diseases such as diabetes, HIV, or stroke, where the vast population offers more opportunities for investigation, low-prevalence or rare diseases can benefit greatly from the application of such methodologies to identify unmet needs or improve the network of care and treatment for patient communities. Therefore, it is critical for public health institutions to systematically explore how to effectively use interactive features on social media to attract public attention and maintain communication with the public.

Further research should also evaluate a qualitative analysis of the selected topics, offering insights that can help improve the judgement in understanding the topic relationships [51].

## 5. Limitations

For all its strengths, this study has limitations. We based our analysis on just one specific day that may not describe all the dimensions and themes about Lupus Awareness Month. Data collection relied on a public Twitter API was able to detect 4434 tweets in English, which may have led to a loss of some tweets.

In the dataset, most accounts were based in the UK or USA due to the language choice. Only a tiny percentage of accounts reported the geographical location, making it impossible to properly explore specific geographic characteristics at the country level. Therefore, future studies could take into account and explore a longer period, consider other languages, and evaluate geographic and ethnic effects that play a role in Lupus.

We used structural topic modeling to analyze tweet texts, while other methods may offer other types of classification based on natural processing language or deep learning suitable for tweet texts [52]. However, despite these limitations, this study provides an extensive and detailed methodological approach offering useful insights into social media platform dynamics regarding Lupus, which is still little investigated.

## 6. Conclusions

Applying the combination of topic modeling and user network analysis, we were able to detect two main types of user communities with specific types of concerns and topic discussions and define different narratives employed by influencers.

The findings of this study provided a detailed example of the implications for healthcare organizations when detecting, understanding, and defining topic discussions and communicative functions available on Twitter. We thus provided an overview of the valuable opportunity to identify appropriate user audiences and share what might be suitable content to engage and interact them, going beyond word frequency, hashtag counts, and online community detection. The importance of considering public health issues involves the complexity embedded in any kind of low-prevalence/rare disease where the low number of patients makes it hard to obtain valuable information, increasing public awareness, and impact on health behavior.

Future research should consider the geographical location and related characteristics of health communication strategies to provide insights able to implement health information dissemination for health practitioners and policymakers.

This type of research can fill the knowledge gap between clinical epistemological uncertainty and patient experiential knowledge when dealing with lupus. We believe that the proposed approach may have a significant role in public health, applying such research indicators and methodologies to aid decision-makers in designing interventions and effective communication strategies.

## Figures and Tables

**Table 1:** Top scored influencers.

Title	Screen Name	Influencer Score	Screen Name	Network Influencer Score
1	Integrated clinical Hospital; USA	35.182	Peter Morley	0.99
2	information boards Blog; UK	26.257	lupusuk	0.66
3	Physiopedia	21.259	Information boards Blog; UK	0.37
4	Newspaper; South Africa	20.830	Advocate page; USA	0.28
5	Radio; Nigeria	12.814	Lupus charity; USA	0.24
6	HibbsLupusTrust	12.271	Charity; UK	0.18

**Table 2:** Most representative tweet texts and topic label selection.

**Learning and sharing (topic 1):** “To anyone with Lupus, it does get better. With time you learn your triggers, you learn to pace yourself and most importantly you learn to listen to your own body.”; “Help us spread awareness for #lupus on #WorldLupusDay!” “Learn more about #lupus brain fog and get tips for coping with it in our article at.”
**Information and advice (topic 2):** “Do eat a healthy, balanced diet try to stay active when you’re having a flare-up try walking or swimming get lots of rest try relaxation techniques to manage stress”; “stress can make symptoms worse.” “For information about available support, please take a look at our article here.”
**Feeling loneliness (topic 3):** “Invisible. For everyone with a disability or an illness that can’t be seen. YOU are not alone, WE are not alone. Today is #WorldLupusDay and we are especially thinking of everyone in the world who has #Lupus #invisibleillness #chronicpain #health #mentalhealth.” “In conjunction of special day for this invisible illness I would like to encourage everybody to appreciate your health and for all Lupus fighter in the world.”
**Spread awareness (topic 4):** “MAY 10 is WORLD LUPUS DAY! Spread Lupus Awareness share the Lupus In Color Butterfly Woman. Spread Lupus Awareness Today!”; “Today is World Lupus Day! Show me your purple! #LupusAwarenessMonth,”; “I chose purple, and you?”
**Social support (topic 5):** “Today around the world #Lupus advocates, patients, and amp; supporters are working hard to spread #LupusAwareness. For #WorldLupusDay we’ll highlight our #LupusChat community members, advocates, caregivers, doctors, and friends who work tirelessly daily to educate others about Lupus.” “Just because something doesn’t directly affect you doesn’t make it irrelevant. Sending out strength and encouragement to everyone battling lupus, extra love to my queen.”
**Advocating (topic 6):** “Government would prefer narcotics or sleep medication, which isn’t natural and addictive but that’s ok they get their money from the big old pharma companies #kickbacks #opioidcrisis but they’re getting paid right?!?”; “#WorldLupusDay; Sen Resolution presented (…) We encourage ALL our legislators to join them.”; “If you think #PreExistingConditions protections aren’t important, remember someone you love could have an accident, that will change how you think about this.”
**Patient stories (topic 7):** “My scars are my war wounds, my proof that I survived. They show me that I am...” “Lupus is a long-term condition causing inflammation to the joints, skin and other organs. There’s no cure, but symptoms can improve if treatment starts early. Read about the symptoms here…”
**Disease description(topic 8):** “#Lupus is a severe + life-changing autoimmune disease that can affect any organ in the body. Yet it is also an illness where “but you don’t look sick” is truly apt as the pain, suffering + heavy duty meds aren’t always visible.”; “Symptoms can flare up and settle down, often the disease flares up (relapses) and symptoms become worse for a few weeks, sometimes longer.” “How lupus is diagnosed? As lupus symptoms can be similar to lots of other conditions, it can take some time to diagnose.”
**Involvement (topic 9):** “Learn more about the disease and how you can get involved with the charity at”; “Let’s Join Together to Fight Lupus! #WorldLupusDay”; “Did you know that over 1:1000 Canadian men, women and children are living with lupus? Let’s join together in the fight against #lupus!”
**Encouraging (topic 10):** “Keep fighting and know we are fighting with YOU!”; “to all the Lupus Warriors still fighting every day. You’re amazing and you’re strong. Keep the faith.”; “To all those living with Lupus around the world, keep fighting and may your efforts to awareness be successful.”
**Body symptoms(topic 11):** “As well as the 3 main symptoms, you might also have: weight loss, swollen glands, sensitivity to light (causing rashes on uncovered skin), poor circulation in fingers and toes (Raynaud’s)”; “#Lupus is a long-term autoimmune disease in which the body’s immune system becomes hyperactive and attacks normal, healthy tissue.”; “The immune system protects the body against infections and diseases. However, in Lupus, the immune system starts attacking the body’s healthy tissue, leading to organ damage and chronic inflammation.”
**Communities effect (topic 12):** “lupus affects approx. 5 million people globally yet there is still a lack of awareness amongst general public and healthcare professionals? On #WorldLupusDay join us in encouraging greater understanding of this condition.”; “Today is #WorldLupusDay. Lupus is a global health problem that affects people of all nationalities, races, ethnicities, genders and ages! There are about 200,000 cases diagnosed in Kenya.”; “Lupus is a global health problem that affects people of all nationalities, races, ethnicities, genders and ages.”

## Appendix A

1. Topic Modeling

The heuristic of the probabilistic topic modeling can be seen in Figure A1.

LDA and other topic models are part of the larger field of probabilistic modeling [1]. Generative probabilistic modeling consider data as arising from a generative process that includes hidden variables. This generative process defines a joint probability distribution over both observed and hidden random variables.

The joint distribution to compute the conditional distribution of the hidden variables is given to the observed variables. This conditional distribution is also called the posterior distribution.

Structural topic modeling extends to the LDA framework. STM allows for correlations among topics. Covariate data including document metadata influences topic prevalence within documents. STM also uses (document-specific) covariate data to define distributions for word use within a topic [2].

We employed the ldatuning package [3] using the log-likelihood method via Gibbs sampling. Specifically, we used the “Griffiths” [4] and “CaoJuan” [5] metrics scores.

2. STM Evaluation

The semantic coherence and exclusivity values were associated with each topic. Numerals represent the average for each model and dots represent topic specific scores.

Each model has semantic coherence and exclusivity values associated with each topic. Figure A4 plots these values and labels each with its topic number.

3. Network Construction

We used a directed graph network G to represent social connections and information flows for Twitter users. In *G* = (*V*, *E*), *V* denotes the set of nodes (Twitter users) and *E* denotes the set of edges (social connections) in *G*. An edge *eij* ϵ *E* corresponds to a set of node pairs (*vi,vj*) that connects node *vi* and *vj* in *G*. To define an edge in the network, we include the lists of users they retweeted. Retweet networks consist of directed links indicating that one user has retransmitted a tweet from another user.

Eigenvector centrality (EC) is a method of computing the approximate importance of each node in a network [6]. The rationale behind this centrality measure is that a node is thought to be more important if it is directly connected to important nodes. This relationship to other highly connected nodes indicates a high level of influence.

The modularity algorithm measures [7] the strength of division of a network into clusters or communities and was applied to detect the number of clusters (communities) in the retweets network.

(A1) $$Q=\frac{1}{2m}\;\underset{i,j}{\sum }\left[A_{ij}-\frac{Ki\;Kj}{2m}\right]\;\delta \left(ci\;cj\right)$$

where *A_ij_* represents the weight of the edge between *i* and *j*, *k_i_* = ∑ *A_ij_* is the sum of the weights of the edges attached to vertex *i*, *C_i_* is the community to which vertex *i* is assigned, the δ function δ (*u*, *v*) is 1 if *u* = *v* and 0 otherwise, and $m=\frac{1}{2}\;\underset{ij}{\sum }A_{ij}$.

4. Gephi Network Parameter Results
